# Detection of bioorthogonal groups by correlative light and electron microscopy allows imaging of degraded bacteria in phagocytes†
†Electronic supplementary information (ESI) available. See DOI: 10.1039/c5sc02905h


**DOI:** 10.1039/c5sc02905h

**Published:** 2015-10-23

**Authors:** Daphne M. van Elsland, Erik Bos, Wouter de Boer, Herman S. Overkleeft, Abraham J. Koster, Sander I. van Kasteren

**Affiliations:** a Division of Bio-organic Synthesis , Leiden Institute of Chemistry , Gorlaeus Laboratories , Leiden University , Leiden , The Netherlands . Email: s.i.van.kasteren@chem.leidenuniv.nl; b Institute for Chemical Immunology , Gorlaeus Laboratories , Leiden University , Leiden , The Netherlands; c Department of Molecular Cell Biology , Section Electron Microscopy , Leiden University Medical Center , Leiden , The Netherlands . Email: a.j.koster@lumc.nl

## Abstract

The interaction between parasites and phagocytic immune cells is a key inter-species interaction in biology. Normally, phagocytosis results in the killing of invaders, but obligate intracellular parasites hijack the pathway to ensure their survival and replication. The *in situ* study of these parasites in the phagocytic pathway is very difficult, as genetic modification is often complicated and, if successful, only allows the tracking of pathogen phagocytosis up until the degradation of the engineered reporter constructs. Here we combine bioorthogonal chemistry with correlative light-electron microscopy (CLEM) to follow bacterial processing in the phagolysosomal system. Labelled bacteria are produced using bioorthogonal non-canonical amino tagging (BONCAT), precluding the need for any genetic modification. The bacterial proteome – even during degradation – was then visualised using a novel CLEM-based approach. This allowed us to obtain high resolution information about the subcellular location of the degrading bacteria, even after the proteolytic degradation of reporter constructs. To further explore the potential of CLEM-based imaging of bioorthogonal functionalities, azide-labelled glycans were imaged by this same approach, as well as active-subpopulations of enzymes using a 2-step activity-based protein profiling strategy.

## Abstract 2

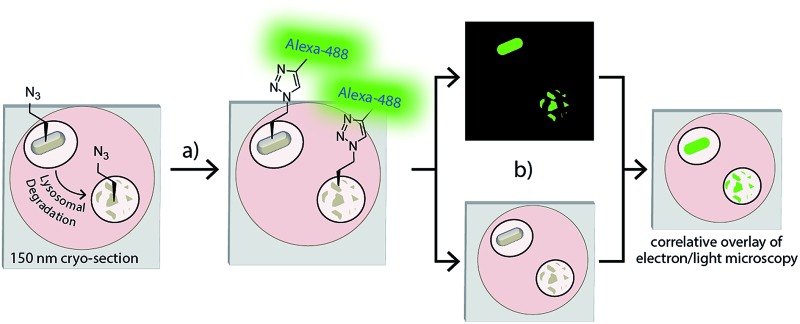
The correlative light-electron microscopy is reported showing the labels in their ultrastructural context.

## Introduction

Phagocytic degradation is a question of great biological relevance, as it is one of the key mechanisms by which the immune system keeps pathogens at bay. As a consequence, subversion of the phagolysosomal pathway is a survival strategy employed by a wide range of parasites, which collectively are responsible for a great amount of human morbidity and mortality.^1^

The interaction between immune cells and pathogenic bacteria is very difficult to study,^2^ as intracellular pathogens can be non-trivial to grow *ex vivo* ^3^ and very difficult to genetically alter. Even in (rare) cases where these bacteria can be genetically modified^4^ the imaging of their encounters with host phagocytes is limited to encounters where successful infection is established. Encounters whereby the pathogens are killed and degraded are difficult to image as the proteolysis that is a hallmark of successful phagocytic maturation^5^ results in the degradation of reporter proteins and epitopes.^6^

Bioorthogonal chemistry is a powerful tool for labelling of (sub)-populations of biomolecules in complex biological systems^7^ and could be employed to circumvent these problems. The approach relies on the introduction of a small, physiologically inert chemical group into a biomolecule of interest that can subsequently be visualised using a selective reaction.^8^ The small size, biological stability of the chemical group, and the wide range of biomolecules that can be labelled with this approach makes this method a valuable part of the biochemist's toolkit.^9,10^

Bolstered by the recent successful imaging of a pathogen inside a host phagocyte through the use of a bioorthogonally modified cell wall component, d-alanine,^11–13^ we envisaged that bioorthogonally labelled bacteria could also be used to image degradation events in host phagocytes. Bioorthogonal non-canonical amino acid tagging (BONCAT)^14,15^ for pan-proteomic incorporation of bioorthogonal groups^16,17^ would allow the labelling of a wide range of bacterial species without the need for genetic modification.^18^ Furthermore, unlike reporter proteins, bioorthogonal groups, such as azides^19,20^ have been shown to be stable in the harsh chemical environments of the phagolysosomal system and should therefore be detectable when extensive proteolysis has occurred.

Information about subcellular localization is of key importance when studying parasite–phagocyte-interactions as movement between organelles may be key to the life cycle of certain parasites.^1,21^ Only transmission electron microscopy (TEM)-based techniques allows the study of these pathogens in their subcellular context, as it provides substructural information on the position of any label/antigen within the cell.^22^ However, in contrast to superresolution imaging,^23,24^ no methods have been reported that allow the visualization of bioorthogonal groups using EM-based approaches.^25^

**Fig. 1:**
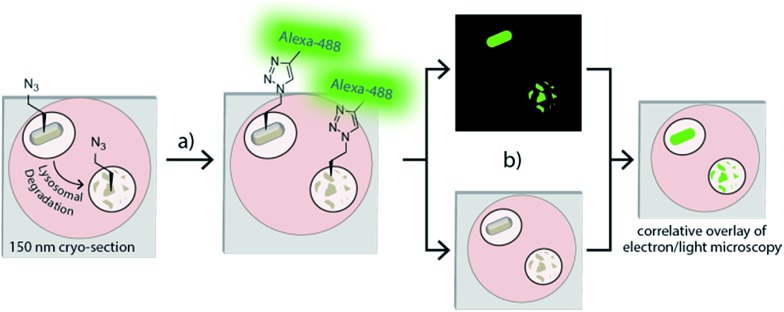
Overview: (a) phagocytosed azido-*E. coli* can be fluorescently visualised in an ultrathin cryosection using a ccHc-reaction with a fluorophore; (b) overlay of this image on an electron micrograph provides an ultrastructural context for the signal with nanometre-resolution. As the bioorthogonal handle is stable to proteolysis, degraded bacteria can be visualized in this manner.

Here we describe the development of a correlative light-electron microscopy (CLEM)-imaging-based visualisation of bioorthogonal groups that allows imaging of BONCAT-labelled bacteria inside phagocytes (Fig. 1); even as they are being degraded. This approach combines the benefits of confocal microscopy – which allows wide-field navigation to areas of interest^26^ – with those of electron microscopy (EM) – which provides narrow field high resolution information about the interior of the cell.^22^ All approaches described here on the model organism *E. coli* are amenable to application to pathogens, which would open new avenues for studying the events leading to bacterial clearance and/or establishment of intracellular residence by intracellular pathogens.

## Results and discussion

### Optimizing the detection of bioorthogonal groups using CLEM

To develop the CLEM-based imaging of bioorthogonal groups, we initially focussed on azides as our bioorthogonal group of choice, as these are the most versatile of the groups in terms of both incorporation methods and available detection reactions.^27,28^ We used the Met-auxotrophic strain B834(DE3) of *E. coli* as our model bacterium, as it can be readily labelled with azides by metabolic replacement of methionine with azidohomoalanine (Aha).^16,29,30^ However, these labelling strategies can be translated to many other non-auxotrophic bacteria and eukaryotes very effectively.^18^

We first optimised Aha incorporation with respect to cell viability, and incorporation levels into the proteome (Fig. S1†). It was observed that extended incubation times resulted in reduced viability (Fig. S2†) and the formation of inclusion bodies (Fig. S3†). This suggests detrimental effects on protein expression and folding of prolonged exposure to Aha.^18,31^ Labelling for 1 h in presence of 4 mM Aha gave robust signal and showed minimal inhibition of viability (Fig. S2†). These conditions were used for all further imaging studies.

**Fig. 2:**
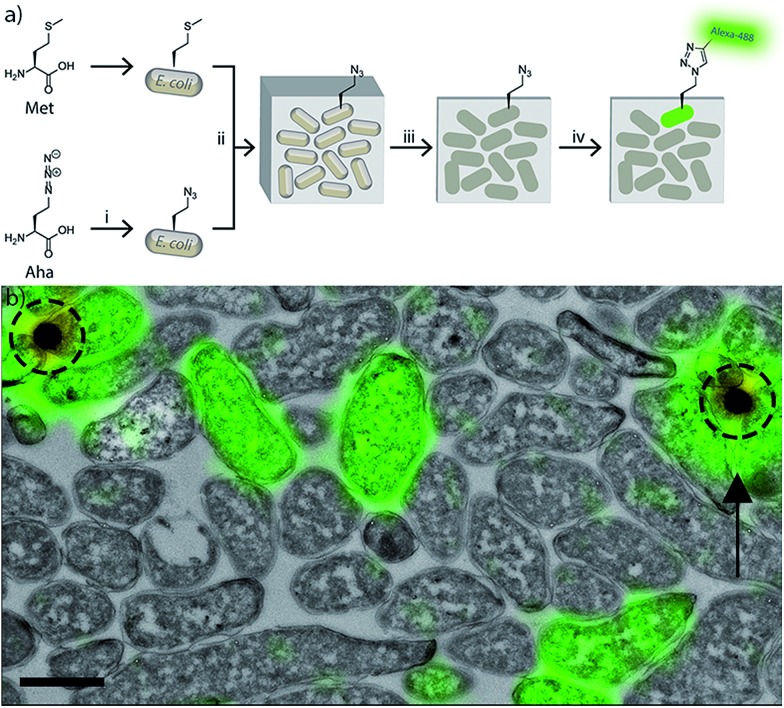
Optimizing CLEM imaging of azido-*E. coli*: (a) azido- (i) and unlabelled wt-*E. coli* were mixed in non-equal ratio (1 : 25) (ii). After Tokuyasu sample preparation and cryosectioning into 75 nm sections (iii), a ccHc-reaction with AlexaFluor-488 was performed (iv). (b) CLEM-image of the experiment (a). Green: AlexaFluor-488 label. Dotted circles: signal from the 100 nm TetraSpeck beads. Scale bar 1 μm.

We focussed our initial development of the on-section labelling of CLEM-samples on cryosections prepared in accordance with Tokuyasu,^32^ as this technique uses mild fixation and sample preparation techniques compatible with azide-chemistry. We prepared sections of mixed azido-*E. coli* and unlabelled wt-*E. coli* at a non-equal ratio to optimize the on-section bioorthogonal labelling (Fig. 2 and S4†).

Of the three available azide-specific bioorthogonal reactions available we focussed on optimising the copper-catalysed Huisgen cycloaddition (ccHc)-reaction^8,33,34^ as it has the lowest background reactivity compared to the strain-promoted cycloaddition reactions, and Staudinger–Bertozzi ligation.^35,36^ Downsides are largely associated with the toxicity of the catalyst.^28^ However, this is not of relevance here as the labelling is performed after aldehyde-fixation and cryosectioning of the samples. We found that glutaraldehyde-free fixation, followed by a blocking step before the ccHc-reaction, combined with ligand-stabilisation of the Cu(i) catalytic species gave the best signal-to-noise levels. The addition of aminoguanidine during the ccHc reduced the detrimental effects of the copper sulfate by-products on the DAPI co-staining.^37^

It was also found that care had to be taken when using copper mounting grids on which the ultrathin sections were placed for CLEM-imaging.

If high concentrations of ascorbate and prolonged reaction times were used for the ccHc, we found that the grids dissolved. This could be prevented by shortening reaction times and keeping the ascorbate concentrations low (<10 mM).

After ccHc-labelling, the sections were first imaged with the confocal microscope (Fig. S4a†) before embedding in methyl cellulose with uranyl acetate. The sections were EM imaged (Fig. S4b†) and correlation of the confocal and EM images was performed using fluorescent electron-dense beads^38^ as fiducials (Fig. 2b and S4c–e†).

### Comparison of GFP-*E. coli* and azido-*E. coli* for imaging phagolysosomal degradation

Most CLEM studies employ the fusion of fluorescent proteins to the protein of interest or antibody-based approaches to allow their identification and localisation.^22^ These labelling approaches have shown to be of great value for the imaging of specific proteins in their cellular context, but only in the cases where genetic modification of the organism has been possible and where the attachment of the fluorescent proteins does not affect protein function.^39^

Immunofluorescence has also been used, but combined with CLEM it either compromises ultrastructure (by virtue of the need of fixation and permeabilisation prior to CLEM-sample preparation),^40^ or suffers from a notoriously low success rate due to compromised epitope availability in samples prepared for TEM.^41^

**Fig. 3:**
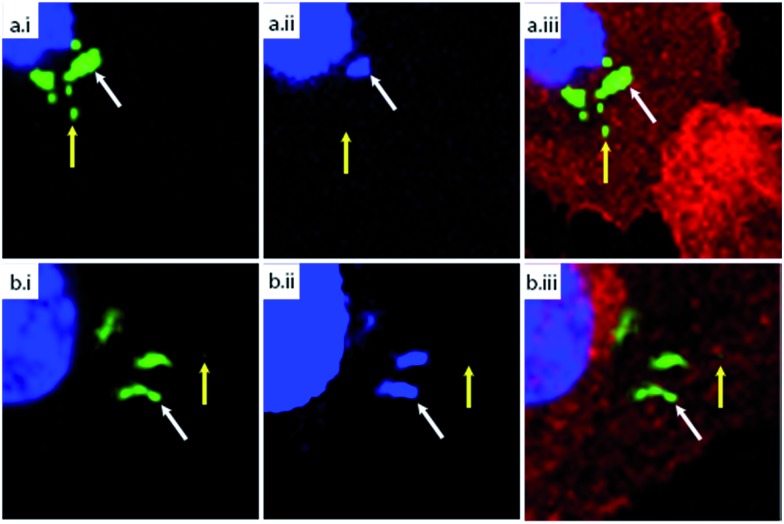
Confocal microscopy of (a) azido-*E. coli* or (b) GFP-*E. coli* after phagocytosis. BM-DCs were pulsed with either azido-*E. coli* or GFP-expressing *E. coli* (45 min pulse). Cells were fixed after a 2 h chase and stained with DAPI (blue), anti-actin (red) and, in case of azido-*E. coli*, AlexaFluor-488 alkyne (green = either GFP or AlexaFluor-488). (i) DAPI/488 nm overlay; (ii) DAPI only; (iii) all fluorescent channels overlay. Yellow arrows indicate a 488-single positive focus, white arrows a DAPI/488 nm double positive focus.

As our main envisaged application of bioorthogonal CLEM imaging was in detecting phagocytosed bacteria during degradation (Fig. 1), we first determined whether our BONCAT-based approach had advantages over genetic methods for the study of these events. The fate of azido-*E. coli* was compared to that of GFP-expressing *E. coli* (Fig. 3, S5 and S6†). We incubated mouse bonemarrow-derived dendritic cells (BM-DCs)^42,43^ with azido-*E. coli* or GFP-*E. coli* for 45 minutes. After washing, the cells were chased for 1 h, 2 h or 3 h prior to fixation, bioorthogonal modification of the azides (where present), and confocal imaging (Fig. 3 and S5†) – time points in which maturation of a phagosome to a phagolysosome are known to take place in these cells.^44^

To assess whether the fluorescent signal originated from an intact or (partially) degraded bacteria, we made use of extra-nuclear DAPI staining: colocalisation of the fluorescent signal with the extra-nuclear DAPI indicates the intactness of the bacterial DNA, which in turn indicates the intactness of the bacterium.^45^ Absence of this colocalisation (*i.e.* 488 nm single positive foci) indicated the degradation of the bacterial genome and thus death.

The azide-based signal persisted significantly more than the GFP-signal after the killing of the bacterium; as indicated by the significantly larger number of DAPI-negative/azide-positive foci at all time points of the chase period compared to DAPI-negative/GFP-positive foci (Fig. S6†). Many of the azide-positive foci were smaller than intact DAPI/azide double positive foci, indicating these signals to originate from partially degraded bacteria. Control samples of wt-*E. coli* were fully negative at all time points (Fig. S5†).

### CLEM-imaging of azido-*E. coli* after uptake by BM-DCs

**Fig. 4:**
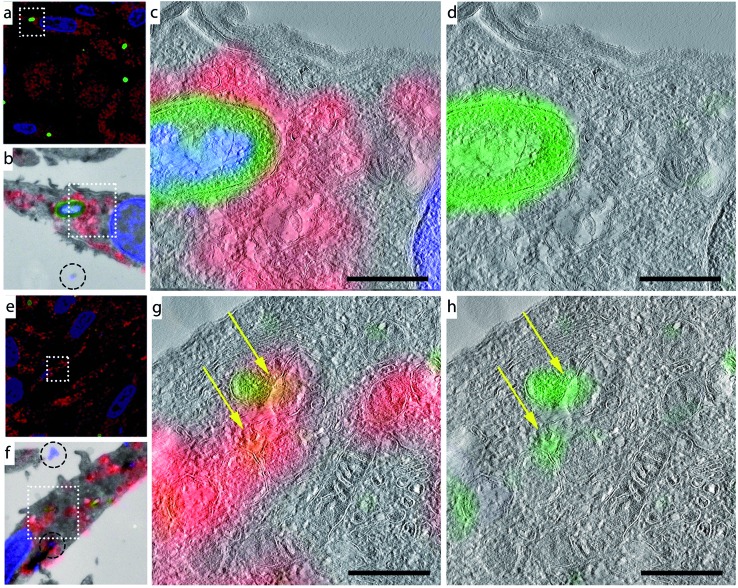
CLEM imaging of phagocytosed azido-*E. coli*: BM-DCs were pulsed with azido-*E. coli* (45 min pulse). Cells were washed with PBS to remove unbound/non-internalized *E. coli*. Samples were fixed immediately after pulsing (a–d) or after a 3 h chase (e–h). Cells were subjected to Tokuyasu sample preparation and cryosectioned into 150 nm sections. Sections were reacted with AlexaFluor-488 alkyne using ccHc-conditions (green), anti-LAMP-1 (red) and DAPI (blue). DAPI staining and blue fiducials (indicated with circles in b and f) were used for correlation purposes. (a/e) Confocal microscopy images; (b/f) CLEM image obtained from overlay LM and EM pictures; (c, d, g and h) CLEM details from (b/f), showing LAMP-1 and 488 nm channels (c/g) or 488 nm alone (d/h). Scale bar 500 nm.

We obtained ultrastructural information about the location of these smaller, DAPI-negative foci by performing CLEM analysis on azido-*E. coli*-treated BM-DCs samples at all four time points (Fig. 4 and S7†). Co-staining with the lysosomal marker LAMP-1 revealed that these degraded fragments only partially resided in LAMP-1-positive late endosomes/lysosomes. This ties in with previous studies showing the existence of a second population of phagosomes in DCs, which do not acidify and never become LAMP-1 positive.^46^ This set of phagosomes has been implicated in DC-specific functions such as cross-presentation.^47,48^

Morphological information obtained from TEM showed that the azide-positive/DAPI-positive foci were intact bacteria, whereas the DAPI-negative foci showed no identifiable bacterial morphology, indicating that this technique allows the imaging of partially degraded bacteria inside mammalian phagocytes.

### Application of CLEM to other bioorthogonal labelling strategies

The above approach highlights the potential of CLEM-imaging in detecting BONCAT-labelled bacteria inside phagocytes. However, bioorthogonal chemistry offers a much broader arena of applications to interrogate specific physiological phenomena.^27^ Two stalwart applications are the labelling of glycans in mammalian cells^49^ and activity-based protein profiling.^50^ To firmly embed our technique within the bioorthogonal chemistry paradigm, we also applied CLEM-imaging in these settings.

**Fig. 5:**
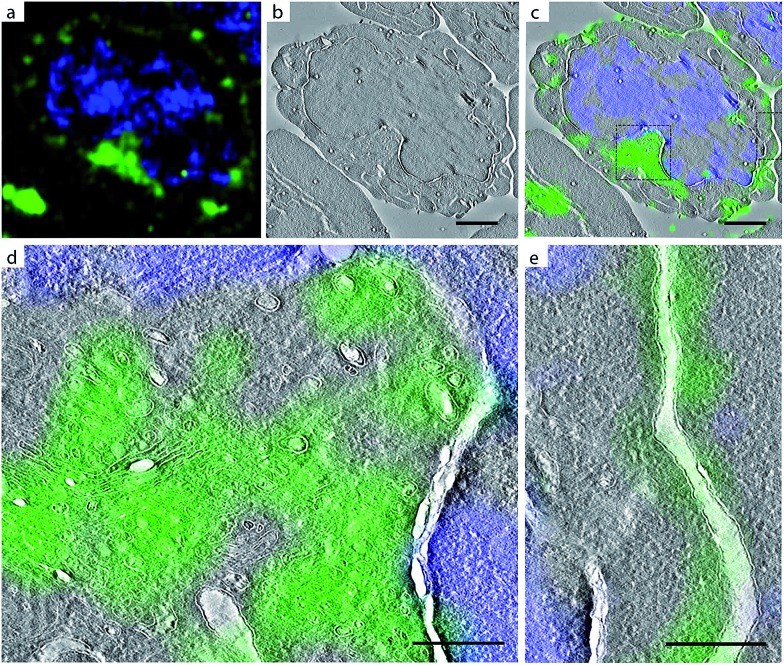
CLEM imaging of bioorthogonal tagged sialylated glycans in Jurkat cells. Green: AlexaFluor-488 alkyne; blue: DAPI. DAPI staining was used for correlation purposes. (a) Confocal microscopy images; (b) EM image; (c) CLEM image obtained from overlay LM and EM pictures; (e) CLEM detail from (c) of intracellular region; CLEM detail from (c) of plasma membrane. Scale bar 2 μm.

We first recapitulated the archetypal imaging of sialic acid-containing glycans on mammalian cells,^49^ by imaging Jurkat cells incubated with Ac_4_-*N*-azidoacetylmannosamine (Ac_4_ManNAz; Fig. S8†), an approach that results in cells with a bioorthogonally labelled sialoglycome.^51^ We subjected these cells to the bioorthogonal labelling and imaging conditions as above and found that LM imaging revealed cell surface and intracellular staining as previously observed by Baskin *et al.* ^52^ (Fig. 5a and S9†). Subsequent correlation with EM-imaging of the same section of the cell (Fig. 5b–e) revealed the labelled sialic acids to reside in the Golgi apparatus (Fig. 5d) and the plasma membrane (Fig. 5e). Incubation with unlabelled control (Ac_4_ManNAc) gave no fluorescent labelling (Fig. S9a†).

We next combined CLEM with two-step activity-based protein profiling.^53,54^ This technique has proven powerful for detecting active sub-populations of *e.g.* serine hydrolases,^50^ cysteine proteases^55,56^ and proteasomes^57^ in a fashion whereby in the first step enzymes are covalently and irreversibly inhibited, after which the reporter moiety is ligated in a second step through bioorthogonal chemistry. One example that was of particular interest to us^58,59^ were the endo-lysosomal cysteine protease family: the cathepsins^55,60^ as these enzymes are not only covalently labelled by a series of analogues of the natural product E64 (ref. 61) and their activity is also restricted to the endo-lysosomal pathway^62^ (although their extracellular activity has also been reported^63^). If their intracellular activity could be pinpointed to LAMP-1 negative vesicles in DCs, this would provide further visual evidence for the degradation of bacteria in LAMP-1-negative phagosomes observed earlier.

As such, their imaging using an azide-modified variant of E64 (DCG-04-azide) will offer an ‘internal standard’ of the selectivity of this approach: the signal of the enzyme is expected to be contained only within membrane-limited vesicles. Furthermore, it should in part overlap with the lysosomal marker LAMP-1.

**Fig. 6:**
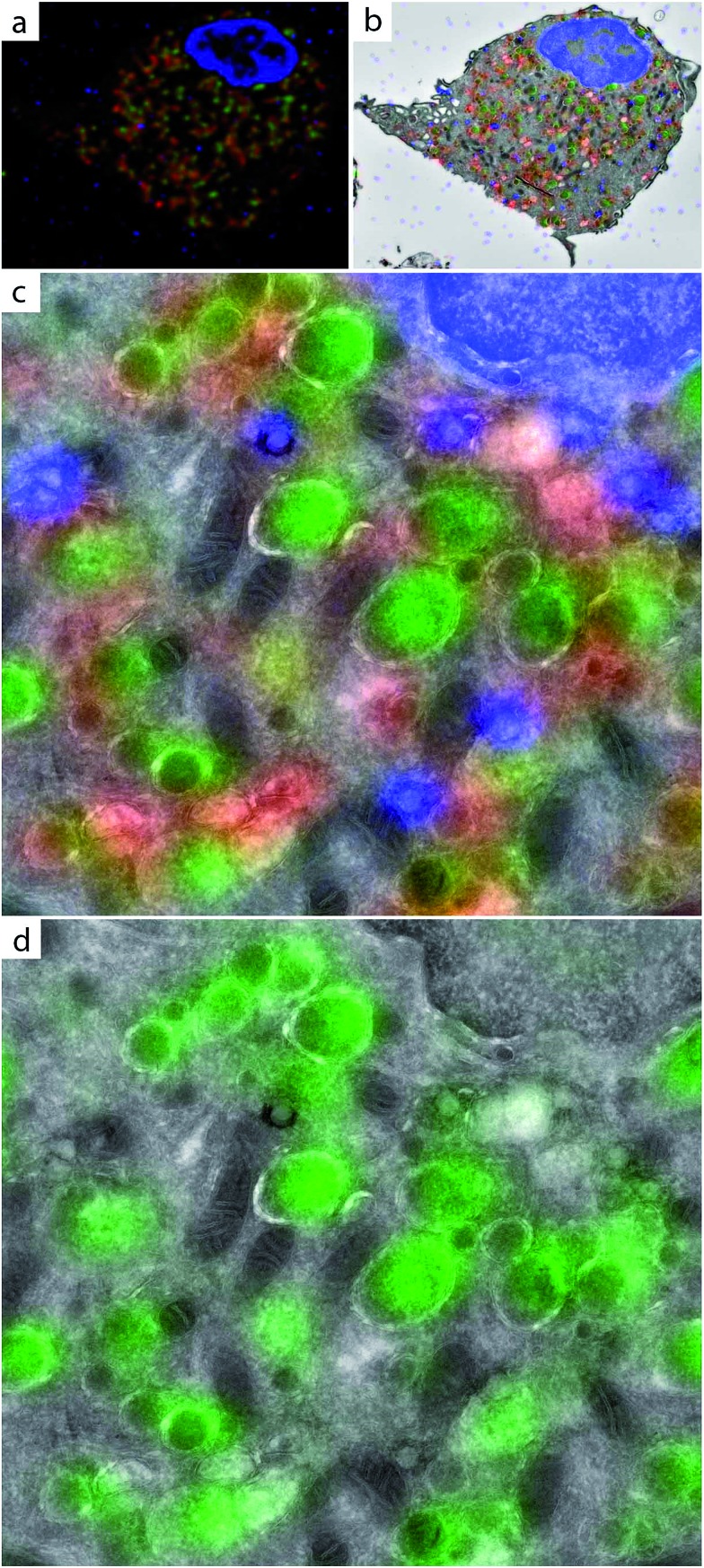
CLEM imaging of active populations of cysteine proteases in BM-DCs. BM-DCs were incubated for 2 h with 10 μM DCG-04-azide (a–d). Cells were fixed in 2% PFA, subjected to Tokuyasu sample preparation and cryosectioned into 150 nm sections. Sections were reacted with AlexaFluor-488 alkyne using ccHc-conditions (green), anti-LAMP-1 (red) and DAPI (blue). DAPI staining and blue fiducials were used for correlation purposes. (a) Confocal microscopy image; (b) CLEM image obtained from overlay LM and EM pictures; (c and d) CLEM details from (b), showing LAMP-1, AlexaFluor-488 and DAPI (c) or only AlexaFluor-488 (d). Scale bar 1 μm.

We incubated BM-DCs with DCG-04-azide using conditions described^64^ and after extensive washing (the 2-step probe is cell permeable and unbound ABP can therefore be removed prior to bioorthogonal labelling and imaging), the cells were fixed, sectioned and subjected to bioorthogonal labelling. The resulting fluorescent signal was found to be fully contained in membrane-limited structures (Fig. 6, S10†) and indeed co-staining with the anti-LAMP-1 antibody revealed a population of vesicles negative for LAMP-1 but with robust cathepsin activity.

These two examples show that CLEM-imaging of bioorthogonal handles is a versatile technique that can be used over a wide range of applications. In future, it will be interesting to see how the structural information will impact the application of activity-based probes for other enzymes and enzyme families, such as the aforementioned serine hydrolases,^65^ and the recently reported protease-specific probes.^66^

## Conclusions

By combining BONCAT of bacteria with CLEM-imaging, we have established a new approach that allowed us to visualise bioorthogonally modified bacteria in an ultrastructural cellular context, even during late stages of bacterial degradation. This approach is of great interest for the study of obligate intracellular parasites that are very hard to study by any other means. As the application of bioorthogonal chemistry is ever expanding, the CLEM-imaging method of bioorthogonal groups described here could also be of great benefit to the study of labelled biomolecules in other fields in which bioorthogonal imaging has proven its value.^25^

Here we demonstrate this value by imaging glycans on the surface and in the Golgi of mammalian cells and by imaging active sub-populations of cathepsins inside the endo-lysosomal pathway. Application of this approach to other bioorthogonal assays (for instance, lipid imaging^67^ and the imaging of newly synthesized proteins^68^), and perhaps in combination with some of the more recently developed bioorthogonal chemistries^69^ will allow the provision of additional structural information to the current imaging methods available for these types of biomolecules.
