# Supplementary material for: Detection of bioorthogonal groups by correlative light and electron microscopy allows imaging of degraded bacteria in phagocytes
Source: Chem Sci. 2015 Oct 23;7(1):752–8. doi: 10.1039/c5sc02905h (PMC5529995; doi:10.1039/c5sc02905h)
Supplement: Supplementary file 1 [file SC-007-C5SC02905H-s001.pdf]

Electronic Supplementary Material (ESI) for Chemical Science.
This journal is © The Royal Society of Chemistry 2015

SUPPLEMENTARY FIGURES

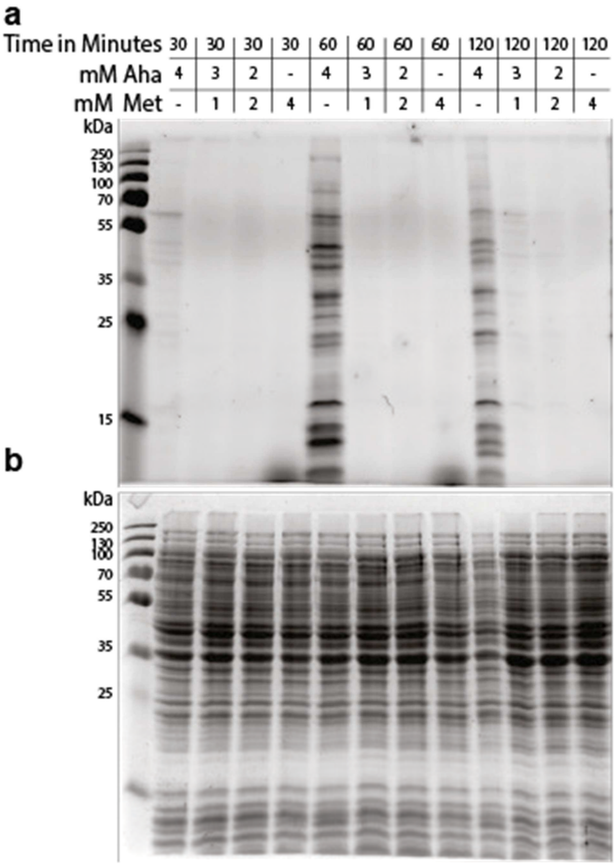

Supplementary figure 1: Aha incorporation in *E.coli* B834: (a) Fluorescence gel of AlexaFluor-488 alkyne labelled *E. coli* cells grown in the presence of the indicated concentrations of Aha and Met. Maximal label incorporation was seen after 60’ in absence of Met and in presence of 4 mM Aha. (b) Coomassie-staining loading control shows relative amounts of total proteins per sample.

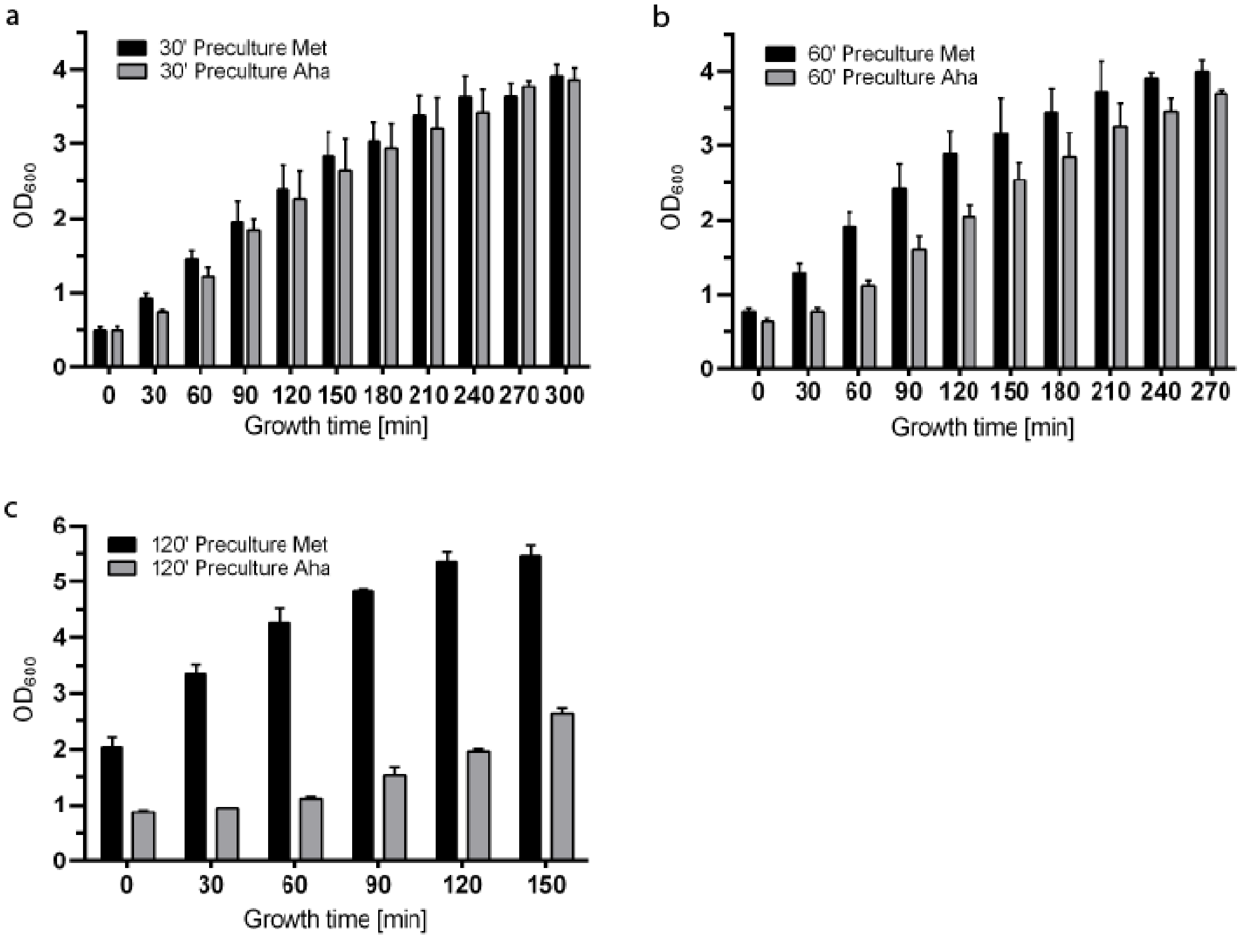

Supplementary figure 2: Outgrowth of *E.coli* B834 that were cultured in the presence of Aha (n=2): *E. coli* B834 cells were grown to an OD_{600} of 0.3-0.5. Cultures were then incubated with 4 mM Aha for either 30’(a), 60’(b) or 120’(c). Cells were then collected and left to grow in LB medium. OD_{600} was measured at indicated time points. Cultures grown in the presence of Aha for t > 60’ showed severe effects on the outgrowth.

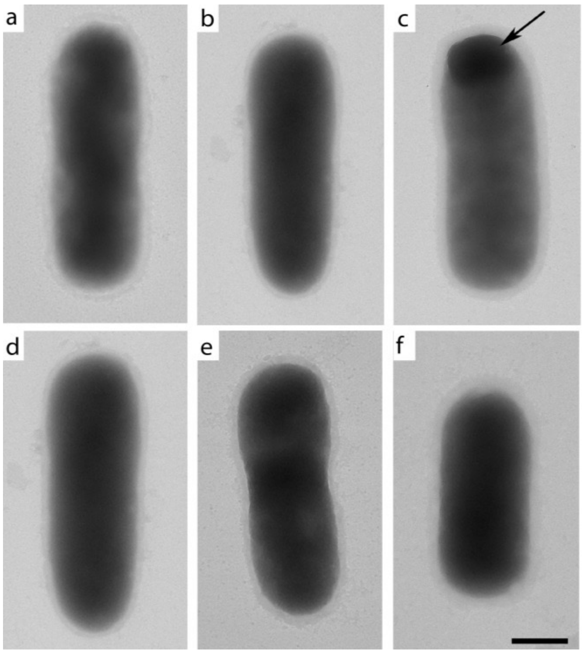

Supplementary figure 3: Inclusion body formation in *E.coli* B834 that were grown in the presence of Aha. *E. coli* B834 cells were grown to an OD_{600} of 0.3-0.5. Cultures were then incubated with 4 mM Aha (a-c), or 4 mM of Met (d-f). After 30'(a, d), 60' (b, e) and 120' (c, f) cells were harvested, fixed with 2% PFA and imaged with the electron microscope. Inclusion bodies were present in *E.coli* cells cultured for 120' in the presence of 4 mM Aha (c). Inclusion body is indicated with an arrow. Scale bar 500 nm.

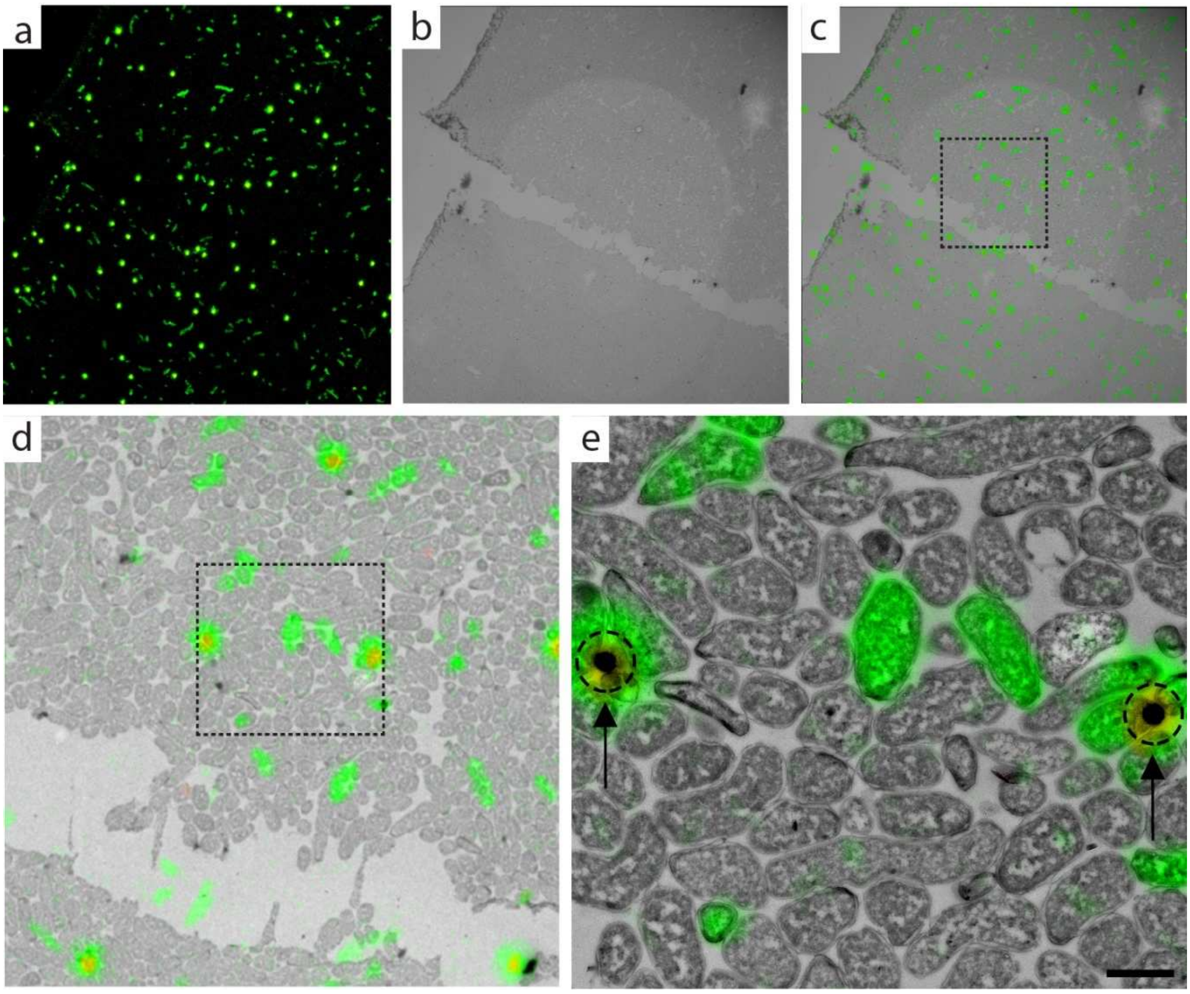

Supplementary figure 4: CLEM image of azido-*E.coli* B834 in a mixture with unlabeled *E. coli* B834 (a) Confocal microscopy image of azido-*E. coli* B834 in a mixture with unlabeled *E. coli* B834. Green = AlexaFluor-488 and 100 nm TetraSpeck beads, Yellow = 100 nm Tetraspeck bead. (b) EM image of azido-*E. coli* B834 in a mixture with unlabeled *E. coli* B834. (c) CLEM image obtained from figure e and f using section shape for correlation. (d) Detail from g. Green = AlexaFluor-488 alkyne and 100 nm TetraSpeck beads, Yellow 100 nm Tetraspeck beads. Tetraspeck beads were used for correlation. (e) Detail from d. Green = AlexaFluor-488 alkyne And 100 nm TetraSpeck beads, Yellow 100 nm Tetraspeck beads (indicated with circles and arrows). Tetraspeck beads were used for correlation. Scale bar 1 µM.

Supplementary figures 5 (Next 3 pages): Confocal imaging of mouse BM-DCs infected with GFP-*E.coli* (a.i-d.iii); Aha-*E. coli* (e.i-h.iii); or Met-*E.coli* (i.i-l.iii). BM-DCs were incubated with *E. coli* cells for 45'. Cells were washed with PBS to remove unbound/non-internalized *E.coli*. After a 45' pulse (a.i-iii/e.i-iii/i.i-iii), 1h chase (b.i-iii/f.i-iii/j.i-iii), 2h chase (c.i-iii/g.i-iii/k.i-iii) or 3h chase (d.i-iii/h.i-iii/l.i-iii) cells were fixed with 4% PFA for 15'. Aha/Met E.coli containing cells were labeled with AlexaFluor-488 alkyne using ccHc- conditions. All cells were stained for actin (red), to outline cellular shape. DAPI staining was performed to mark the DNA in both *E. coli* and BM-DCs. Green = GFP (a.i-d.iii)/AlexaFluor-488 (e.i-l.iii),

Red = Actin, Blue = DNA DAPI stain. White arrows indicate colocalization of DAPI signal and GFP/azide signal respectively. Yellow arrows indicate lack of DAPI colocalization of GFP/azide positive signals. Anti-actin-Alexa647 was used to visualise cellular boundaries.

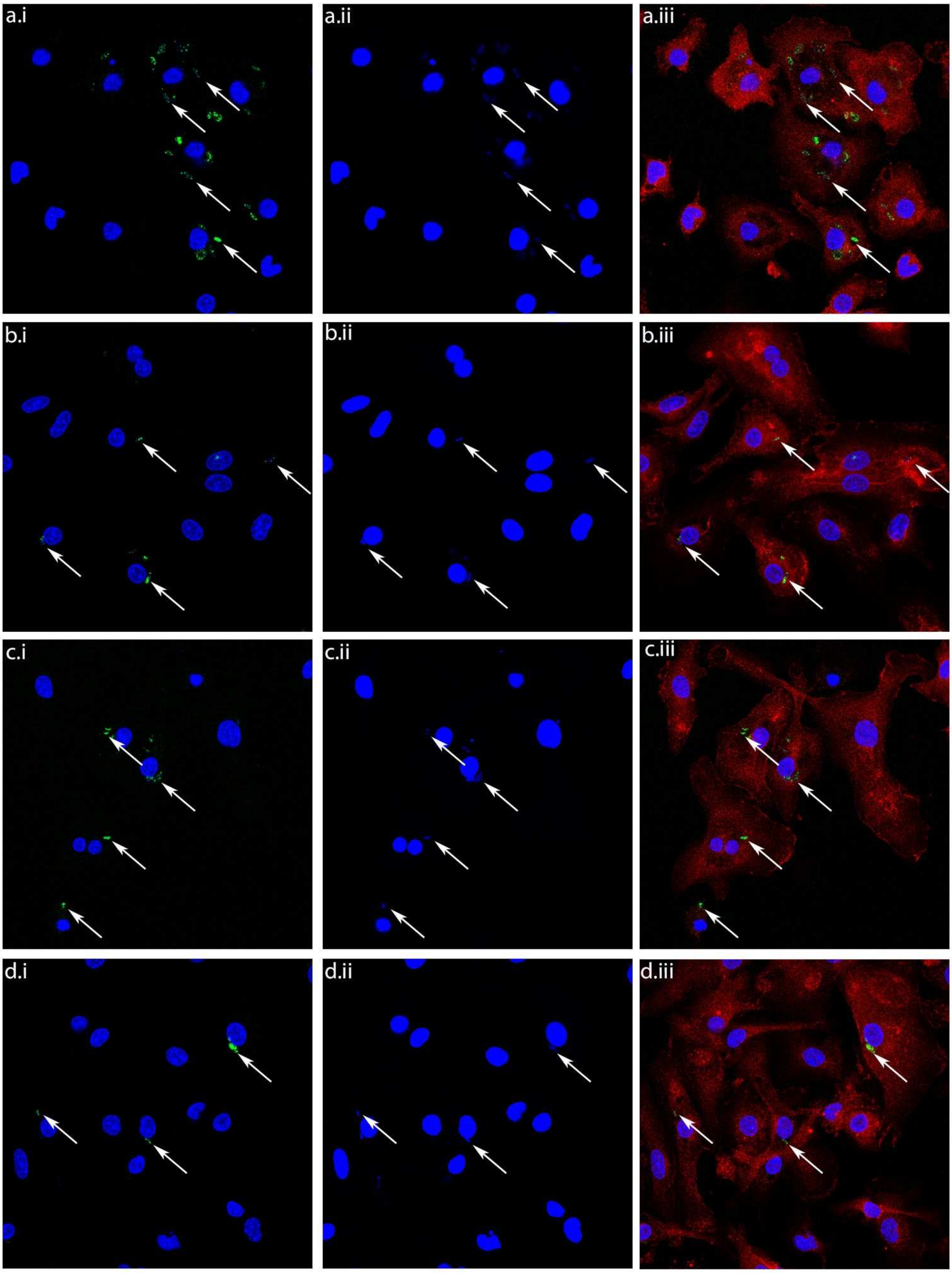

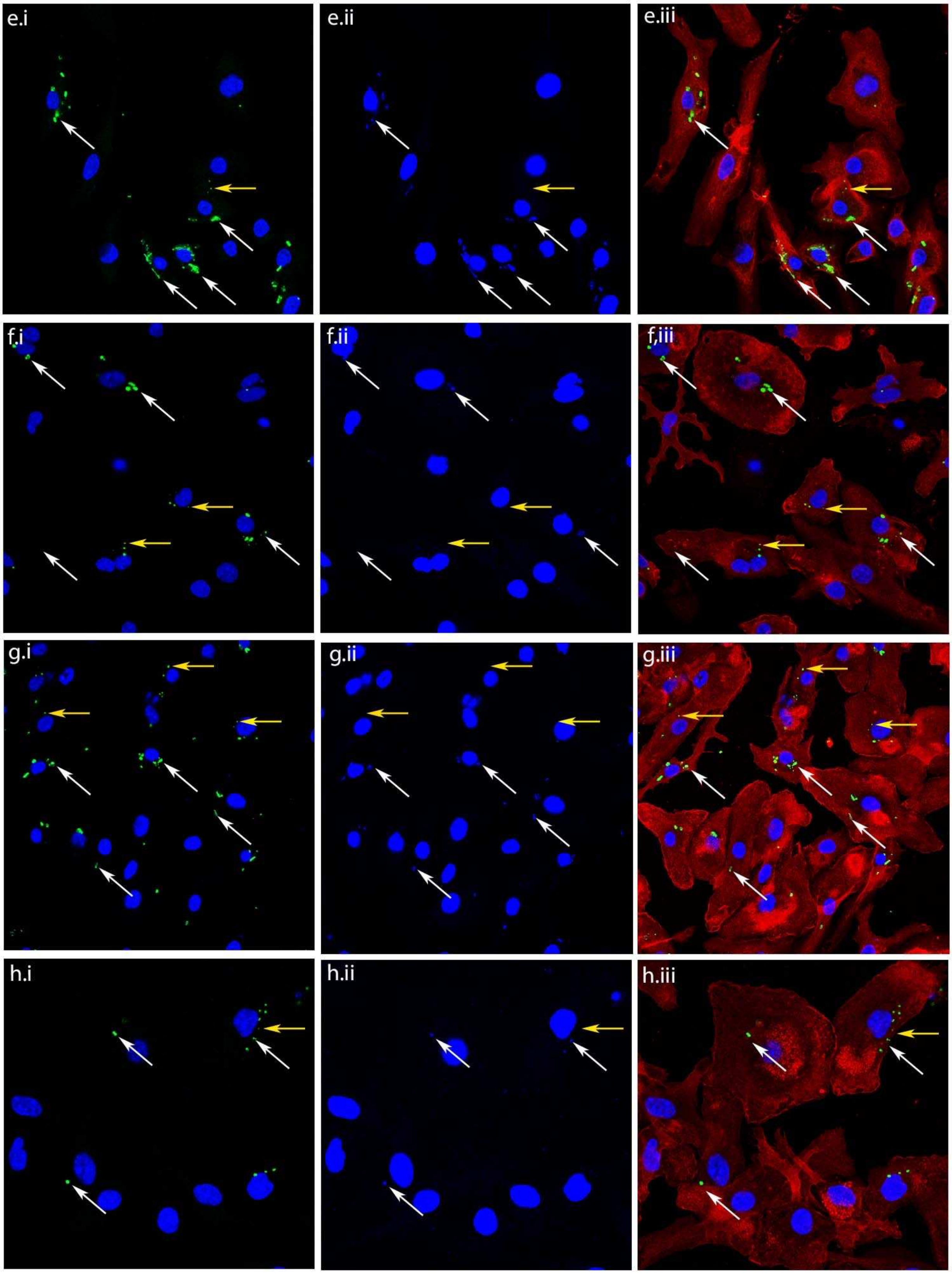
e.i
e.ii
e.iii
f.i
f.ii
f,iii
g.i
g.ii
g.iii
h.i
h.ii
h.iii

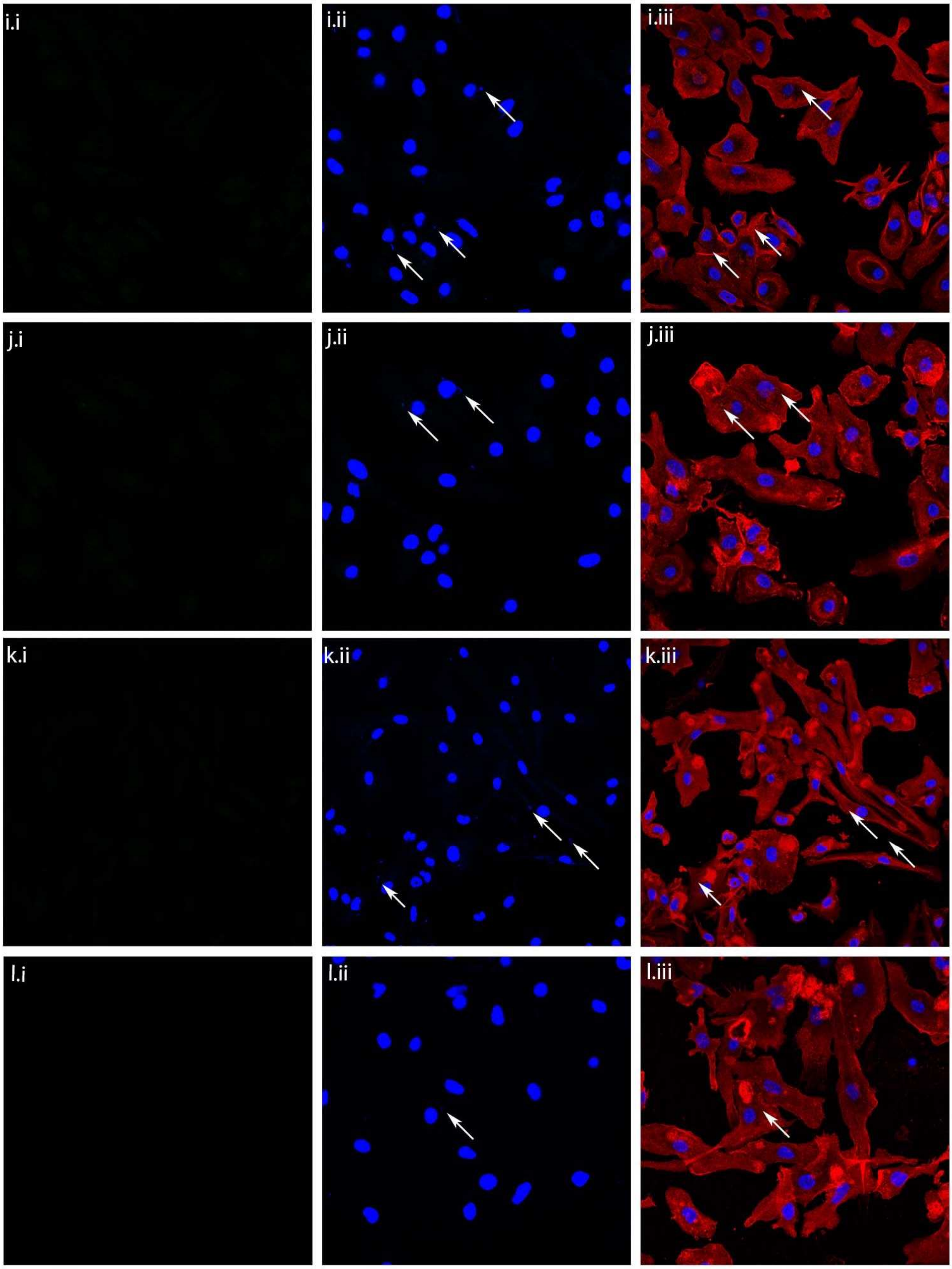
i.i
i.ii
i.iii
j.i
j.ii
j.iii
k.i
k.ii
k.iii
l.i
l.ii
l.iii

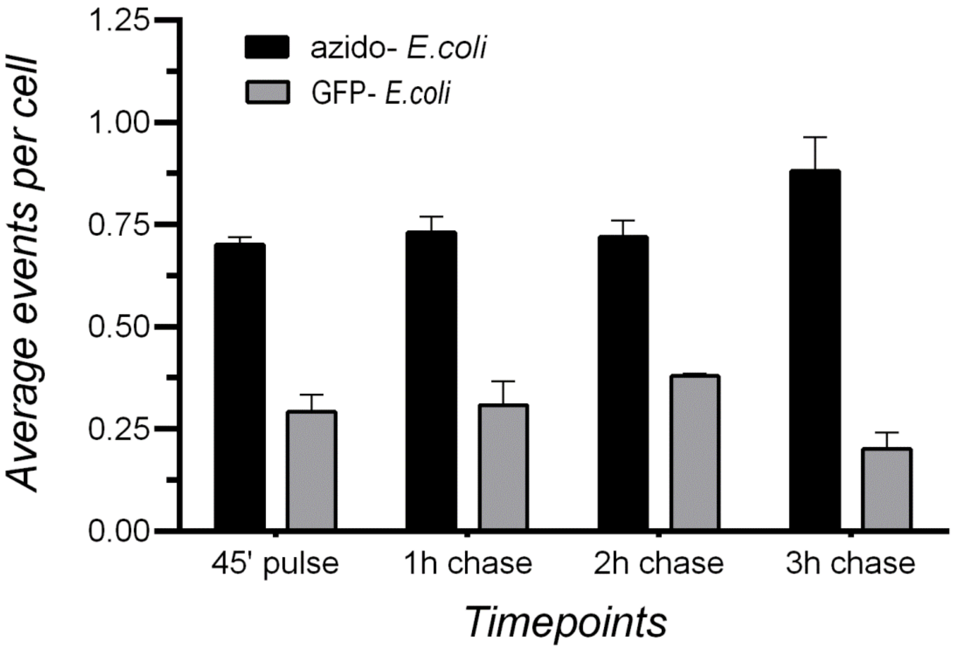

Supplementary figure 6: Comparison of GFP-E. coli and azido-E. coli for imaging phagolysosomal degradation in BM-DCs. BM-DCs were incubated with either GFP-E. coli or azido-*E.coli* cells for 45'. Cells were washed with PBS to remove unbound/non-internalized *E.coli*. After a 45' pulse, 1h chase, 2h chase or 3h chase cells were fixed with 4% PFA for 15'. Azido-*E.coli* containing cells were labeled with AlexaFluor-488 alkyne using ccHc- conditions. From each condition confocal microscopy pictures were made (Fig. S5a-h). Based on these pictures, the average number of green foci, that represented degraded bacteria per cell, was determined. Only the foci were counted that were within the focus plane of the cell (determined by the actin staining) and had no overlap with extra-nuclear DAPI-stain. Per condition 50 cells were counted. N=2.

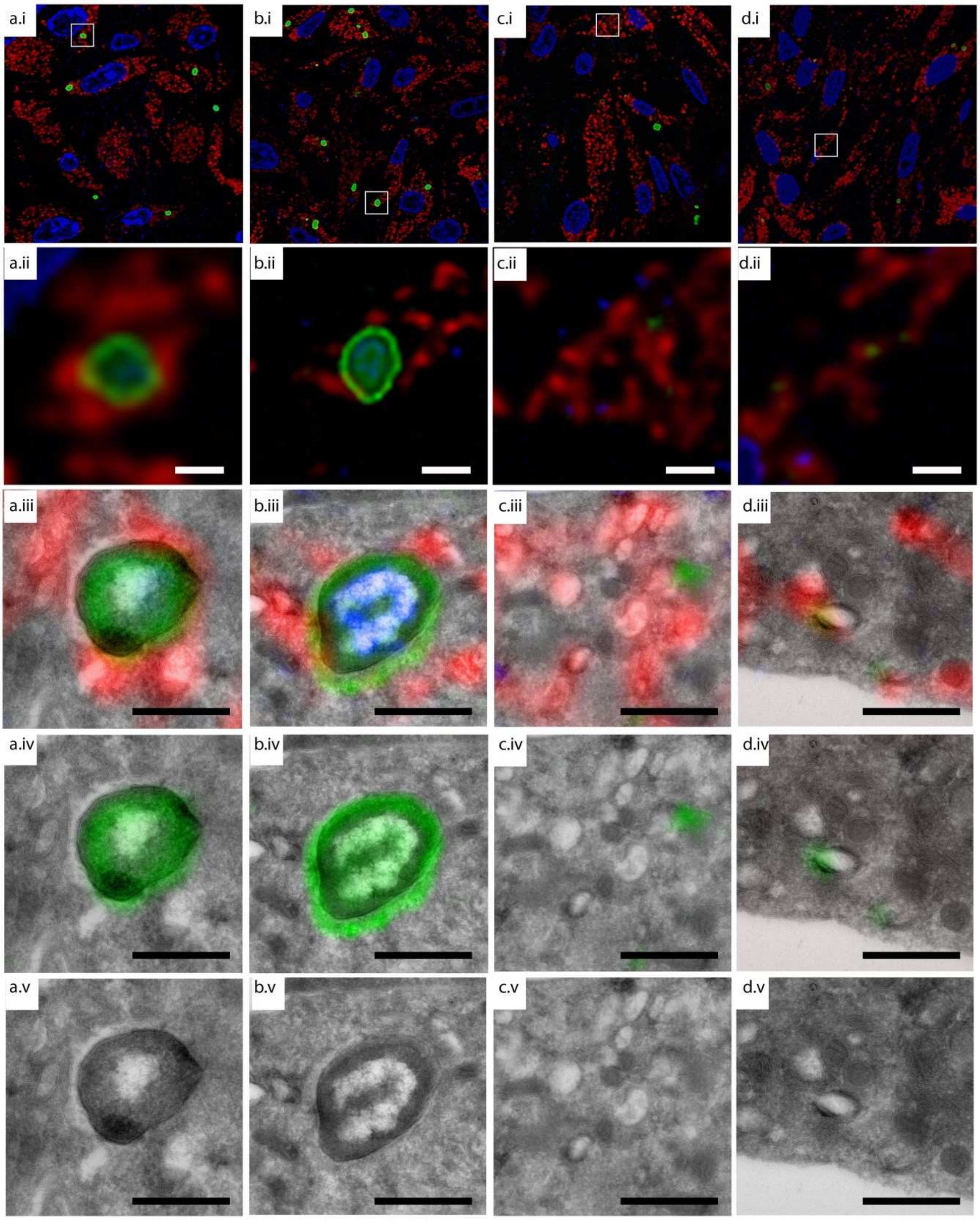

Supplementary figure 7: CLEM imaging of mouse BM-DCs infected with azido-*E.coli*. BM-DC's were incubated with *E. coli* cells for 45'. Cells were washed with PBS to remove unbound/non-internalized *E.coli*. After a 45' pulse (a.i-v), 1h chase (b.i-v), 2h chase (c.i-v) or 3h chase (d.i-v) cells were fixed in 2% PFA, subjected to Tokuyasu sample preparation (including gelatin embedding and sucrose infiltration) and crysectioned into 150 nm sections. Sections were reacted with AlexaFluor-488 (green) alkyne using ccHc- conditions, anti-LAMP1 (red) and DAPI (blue). DAPI staining and blue fiducials were used for correlation purposes. (a.i-d.i) Confocal microscopy image of bioorthogonal tagged *E. coli* B834 incubated with mouse BM-DCs (a.i) 45' pulse (b.i) 1h chase (c.i) 2h chase (d.i) 3h chase. Green = AlexaFluor-488, Red = LAMP1, Blue = nuclear DAPI stain and fiducial beads. (b) Detail from a. (c) b-CLEM image obtained from overlay EM picture and figure b, using blue fiducials and DAPI stain for correlation.

(d) Similar detail from a.iii-d.iii without red and blue signal. (e) Similar detail from a.iii-d.iii EM image only. Scale bar 500 nm.

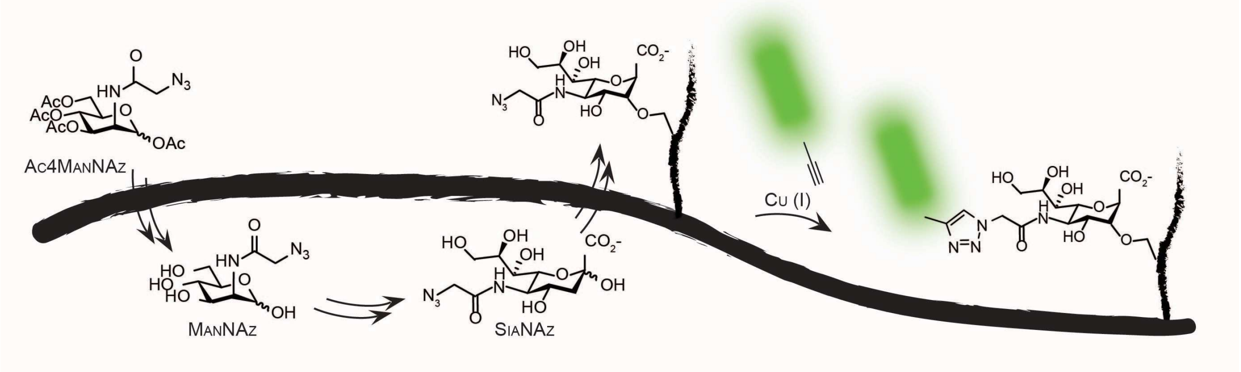

Supplementary figure 8: Scheme for metabolic labeling of sialylated glycans. Peracetylated N-azidoacetylmannosamine (Ac4ManNAz) enters the cell, after which its acetyl groups are cleaved of. ManNAz then passes through the steps of the sialic acid biosynthetic pathway and is converted to azido sialic acid (SiaNAz). After conversion to the nucleotide sugar CMP-SiaNAz, SiaNAz is incorporated into cell-surface glycoconjugates by sialyltransferases. Azido tagged glycans were subsequently labeled with AlexaFluor-488 alkyne via a ccHc on Tokuyasu sections of the cell samples.

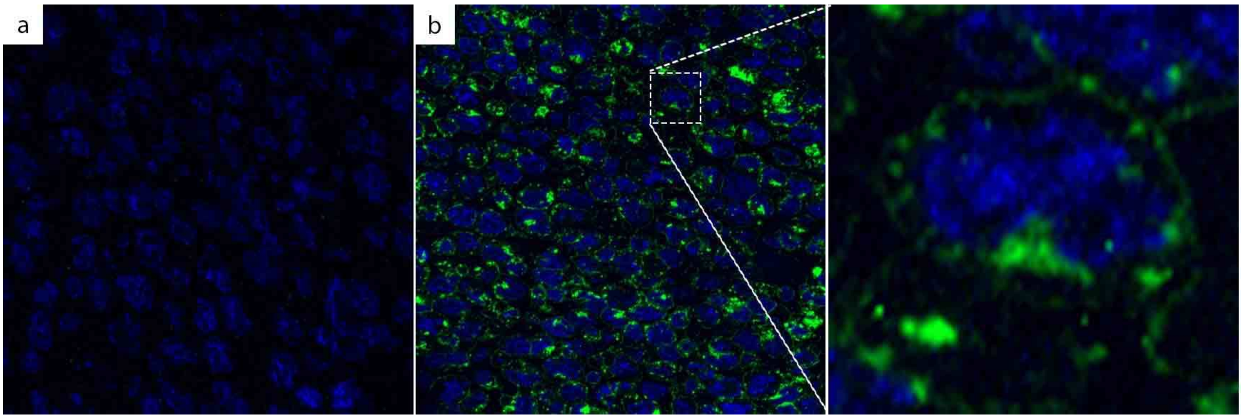

Supplementary figure 9: Confocal image of on section labeling of bioorthogonal tagged sialylated glycans in Jurkat cells. Cells were incubated for 72 h with 50 µM of Ac4ManNAc (a) or Ac4ManNAz (b). Cells were fixed in 2% PFA, subjected to Tokuyasu sample preparation and crysectioned into 150 nm sections. Sections were reacted with AlexaFluor-488 alkyne using ccHc- conditions and DAPI. Green = AlexaFluor-488, Blue = nuclear DAPI stain.

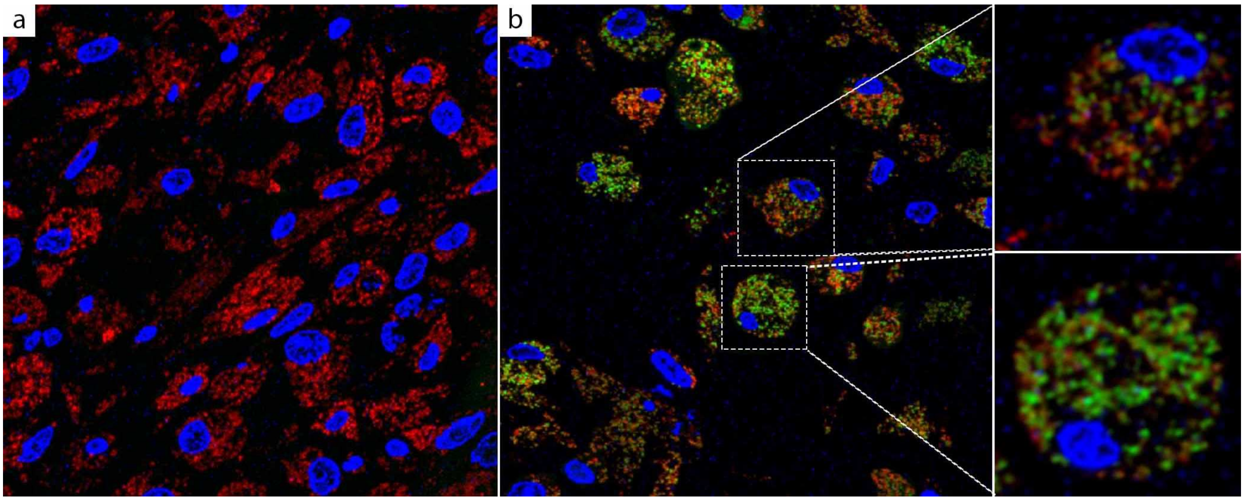

Supplementary figure 10: Confocal image of on section labeling of BM-DCs incubated with DCG-04 that targets active cysteine proteases. Cells were incubated for 2h with 10 µM of DCG-04-Amine (a) or DCG-04-azide (b). Cells were fixed in 2% PFA, subjected to Tokuyasu sample preparation and crysectioned into 150 nm sections. Sections were reacted with AlexaFluor-488 alkyne using ccHc-conditions, anti-LAMP1 and DAPI. Green = AlexaFluor-488, Red = LAMP1, Blue = nuclear DAPI stain and fiducial beads.

MATERIALS AND METHODS

E. coli culturing conditions and growth measurements

E. coli B834 were grown overnight at 37 °C in Lysogeny Broth (LB) medium. The following day cultures were diluted 1:50 in LB medium and grown at 37 °C till an OD_{600} between 0.3-0.5. Subsequently cells were collected and resuspended in Selenomet medium (Molecular Dimensions) and supplemented with different concentrations of either Azidohomoalanine (Aha) (Bachem) or Methionine (Met) (Sigma-Aldrich). After 30', 1h, 2h and 3h OD_{600} were measured and cells were collected by centrifugation for further analysis and BM-DC infection experiments. To monitor the outgrowth of *E. coli* cells that were cultured in the presence of Aha/Met, cells were collected upon centrifugation at the indicated time points after which Aha/Met containing medium was replaced for LB medium and OD_{600} measurements were performed.

E. coli B834 GFP_{A206K} was grown overnight at 37 °C in LB medium. The following day cultures were diluted 1:50 in LB medium and grown at 37 °C till an OD_{600} between 0.3-0.5. Throughout culturing, cultures were supplemented with 100 µg/ml Ampicillin. The vector pRD35 for the constitutive expression of GFP_{A206K}, was constructed by cloning GFP into pUC21 using NsiI and MluI restriction sites. An A206K mutation was introduced by site directed mutagenesis PCR to prohibit dimerization of GFP[1]. The constitutive hns promoter and ribosomal binding site (the 258 bases upstream of the *E. coli hns* gene), were amplified by PCR, using *E.coli* K12 as a template, and positioned upstream of GFP by way of XhoI and NsiI. Used primers are indicated in table 1.

GFP fw NsiI	ACA-ATG-CAT-AGT-AAA-GGA-GAA-GAA-CTT-TTC-ACT-GGA-GTT-G
A206K fw	CCT-GTC-CAC-ACA-ATC-TAA-ACT-TTC-GAA-AGA-TCC-C
A206K rev	GGG-ATC-TTT-CGA-AAG-TTT-AGA-TTG-TGT-GGA-CAG-G
GFP R	CAC-ACG-CGT-TTA-TTT-GTA-TAG—TTC-ATC-CAT-GCC-ATG-TGT-AAT-CC
HNS-Chrom-Fw-XhoI	GAA-CTC-GAG-GGT-CGT-CAG-CCT-ACG-ATA-ATC-TCC-CC
HNS-Chrom-Rev-NsiI	ACT-ATG-CAT-TCT-AGT-AAT-CTC-AAA-CTT-ATA-TTG-GGG-TGG-TTT-G

***Table 1**: Primers used for GFP-plasmid construction*

Inclusion body analysis

At the indicated time points *E.coli* cells were fixed for 2h with 2% paraformaldehyde (PFA) in 0.1 M phosphate buffer. The fixed cells were harvested by centrifugation (13,000 x g for 1 min), and resuspended in PBS. Formvar-carbon coated copper grids were floated on small drops of fixed *E. coli* cells for 5 min at room temperature. Grids were then washed on 3 drops of PBS and 10 drops of aquadest. Cells were imaged with an Tecnai 12 transmission electron microscope (FEI) at 120 kV acceleration voltage.

Mammalian cells and culture conditions

Mouse bone marrow derived dendritic cells were generated from B57BL/6 mice bone marrow essentially as described[2] with some modifications. Briefly, bone marrow was flushed from femurs and tibia and cells were cultured in IMDM (Sigma Aldrich) supplemented with 8% heat-inactivated fetal calf serum, 2mM L-glutamine, 20µM 2-Mercaptoethanol (Life Technologies), penicillin 100 I.U./mL and streptomycin 50µg/mL in the

presence of 20ng/mL GM-CSF (ImmunoTools). Medium was replaced on day 3 and 7 of culture and the cells were used between days 10 and 13.

E. coli B834 cells were added to the BM-DCs as suspensions in PBS in a ratio of approximately 25:1, respectively. After 45' of incubation unbound/non-internalized *E.coli* cells were washed off (2x PBS) and medium was replaced. At the indicated time points cells were subjected to confocal microscopy or Tokuyasu sample preparation.

DCG-04-azide or DCG-04 amine[3] were added in a final concentration of 10 µM to the BM-DCs for 2 hours after which the cells were washed with PBS and kept for 16 hours in fresh medium. Subsequently cells were subjected to Tokuyasu sample preparation.

Jurkat cells were grown in RPMI 1640 medium supplemented with 10% heat-inactivated fetal calf serum, 2mM L-glutamine, penicillin 100 I.U./mL and streptomycin 50µg/mL. Jurkat cells were incubated for 3 days with 50 µM of *N*-azidoacetylmannosamine (MannAz)(Invitrogen) or *N*- acetylmannosamine (MannAc)(Sigma-Aldrich) from stock solutions in DMSO.

SDS-PAGE Analysis

At the indicated time points *E. coli* B834 were collected and cells were lysed with lysis buffer (50 mM HEPES pH 7.3, 150 mM NaCL and 1% NP-40) and incubated on ice for 1 h. Subsequently protein concentrations were determined with a Quibit 2.0 fluorimeter (Life Technologies) after which 20 µg of the protein was incubated for 1h with ccHc-cocktail (0.1 M HEPES pH 7.3, 1 mM $CuSO_4$, 10 mM sodium ascorbate, 1 mM THPTA ligand, 10 mM amino-guanidine, 5 µM AlexaFluor-488 Alkyne (Invitrogen)). Samples were then resuspended in 4x SDS Sample buffer (250 mM TrisHcl pH 6.8, 8% w/v SDS, 40% glycerol, 0.04% w/v broomephenolblue, 5% 2-mercaptoethanol) and incubated at 100 °C for 5 minutes. After the samples were run through a Hamilton syringe multiple times to shear the genomic DNA, samples were subjected to SDS-PAGE. Gels were then directly imaged with a Biorad Universal Hood III for in-gel visualization of the fluorescent labeling. As a loading control gels were stained with Coomassie Brilliant Blue. PageRuler Plus Prestained Protein Ladder (Thermo Scientific) was used as a protein standard.

Whole cell confocal microscopy

BM-DCs were seeded (7 x 10^4) on a 12 well removable chamber slide (Ibidi) and left to grow O/N. The following day *E. coli* B834 cells harboring either GFP/Aha/Met were added to the BM-DCs as suspensions in PBS in a ratio of approximately 25:1, respectively. After 45' of incubation unbound/non-internalized *E.coli* cells were washed off (2x PBS) and medium was replaced. At the indicated time points cells were fixed in 4% PFA for 15 minutes. Until further analysis cells were kept in PBS at 4 °C. When all slides were collected, fixed cells were incubated for 30' with blocking buffer (1 % BSA, 1% gelatin cold water fish skin,), for 1h with click cocktail ((0.1 M HEPES pH 7.3, 1 mM $CuSO_4$, 10 mM sodium ascorbate, 1 mM THPTA ligand, 10 mM amino-guanidine, 5 µM AlexaFluor-488 Alkyne (Invitrogen)), O/N with anti actin antibody (abcam), 1h with goat anti rabbit alexa 568 (Invitrogen) and DAPI. After the staining procedures chambers were removed and cells were covered with a small drop of 50% glycerol after which a coverslip was mounted over the grid. Coverslips were fixed using Scotch Pressure Sensitive Tape. Samples were imaged with a Leica TCS SP8 confocal microscope (63x oil lens, N.A.=1.4).

Bioorthogonal labeling on cryo sections

Samples were prepared for cryo sectioning as described elsewhere[4]. Briefly, *E. coli* cells, BM-DCs and Jurkat cells were fixed for 24h in freshly prepared 2% PFA in 0.1 M phosphate buffer. Fixed cells were embedded in 12% gelatin (type A, bloom 300, Sigma) and cut with a razor blade into 0.5 mm^3 cubes. The sample blocks were infiltrated in phosphate buffer containing 2.3 M sucrose for 3h. Sucrose-infiltrated sample blocks were mounted on aluminum pins and plunged in liquid nitrogen. The frozen samples were stored under liquid nitrogen.

Ultrathin *E.coli* cell sections of 75 nm were obtained as described elsewhere. Briefly, the frozen sample was mounted in a cryo-ultramicrotome (Leica). The sample was trimmed to yield a squared block with a front face of about 300 x 250 µm (Diatome trimming tool). Using a diamond knife (Diatome) and antistatic devise (Leica) a ribbon of 75 nm thick sections was produced that was retrieved from the cryo-chamber with a droplet of 1.15 M sucrose containing 1% methylcellulose. Obtained sections were transferred to a specimen grid previously coated with formvar and carbon Grids were additionally coated as indicated with either 100 nm TetraSpeck beads or 100 nm FluoroSpheres (blue) carboxylate-modified (350/440) (Life Technologies).

Samples of the BM-DCs and Jurkat cells were sectioned into a ribbon of 150 nm thick sections using similar methods as described above. Thicker sections showed to improve the section integrity, leading to improved morphology results. To further improve the structural integrity of the ultrathin cryo sections we used a novel micromanipulator (Manip, Diatome) that was mounted on the cryo chamber of the ultramicrotome. This device facilitated section retrieval from the cryochamber and resulted in less overstretching of the sections during thawing[5].

Sections were labeled as follows; thawed cryo sections on an EM grid were left for 30 minutes on the surface of 2% gelatin in phosphate buffer at 37 °C. Subsequently grids were incubated on drops of PBS/Glycine and PBS/Glycine containing 1 % BSA. Grids were then incubated on top of the ccHc- cocktail (0.1 M HEPES pH 7.3, 1 mM $CuSO_4$, 10 mM sodium ascorbate, 1 mM THPTA ligand, 10 mM amino-guanidine, 5 µM AlexaFluor-488 Alkyne (Invitrogen) for 1h and washed 6 times with PBS. Sections containing BM-DCs and Jurkat cells were then labeled with DAPI (1:5000 in PBS for 5 min), and additionally washed with PBS and aquadest. In case of additional immunogold labeling against Alexa 488 grids were subjected to the following steps directly after the ccHc reaction. Grids were washed 5 times with PBS/glycine and blocked again with PBS/Glycine containing 1 % BSA after which the grids were incubated for 1h with PBS/Glycine 1 % BSA supplemented with a rabbit IgG anti Alexa 488 antibody (Invitrogen). After washing with PBS/Glycine and blocking with PBS/Glycine 0.1 % BSA, grids were incubated 20 min on PBS/Glycine 1 % BSA supplemented with protein A coated 15 nm goldparticles (CMC, Utrecht University). Grids were then washed with PBS, fixed with 1% glutaraldehyde in PBS and washed with aquadest.

In case of additional immune-labeling against LAMP-1 grids were subjected to the following steps directly after the ccHc reaction. Grids were washed 5 times with PBS/glycine and blocked again with PBS/Glycine containing 1 % BSA after which the grids were incubated for 1h with PBS/Glycine 1 % BSA supplemented with a rat anti mouse LAMP-1 (Biolegend). Sections were subsequently washed 6 times with PBS, labeled with DAPI (1:5000 in PBS for 5 min), were after washed again with PBS and aquadest.

<u>Microscopy and correlation</u>

Grids containing the sample sections were washed with 50% glycerol and placed on glass slides (pre- cleaned with 100% ethanol). Grids were then covered with a small drop of 50% glycerol after which a coverslip was mounted over the grid. Coverslips were fixed using Scotch Pressure Sensitive Tape. Samples were imaged with a Leica TCS SP8 confocal microscope (63x oil lens, N.A.=1.4). Confocal microscopy was used as it allowed to make image stacks from the sections at different focus planes, this was convenient as the sections were found to be in different focus planes whilst placed between the glass slides and coverslip. Confocal stacks were deconvolved with theoretical point spread functions using Huygens Essential deconvolution software (SVI, Hilversum, Netherlands). After fluorescence microscopy the EM grid with the sections was remove from the glass slide, rinsed in distilled water and incubated for 5' on droplets of unranylacetate/methylcellulose.

Excess of uranylacetate/methylcellulose was blotted away and grids were air-dried. EM imaging was performed with an Tecnai 12 Biotwin transmission electron microscope (FEI) at 120 kV acceleration voltage. Tilt series for electron tomography were collected using Xplore3D (FEI Company) software. The angular tilt range was set from -60˚ to 60˚ with 2˚ increments, and an objective lens defocus of -2 µm at a magnification of 20 K (pixel size is 1 nm). Alignments of the tilt series and weighted-back projection reconstructions for tomography were performed using IMOD[6].

Correlation of confocal and EM images was performed in Adobe Photoshop CS6. In Adobe Photoshop, the LM image was copied as a layer into the EM image and made 50 % transparent. Transformation of the LM image was necessary to match it to the larger scale of the EM image. This was performed via isotropic scaling and rotation. Interpolation settings; bicubic smoother. Alignment at low magnification was carried out with the aid of nuclear DAPI staining in combination with the shape of the cells, at high magnification alignment was performed using the fiducial beads[7].

References

1. D. A. Zacharias, J. D. Violin, A. C. Newton and R. Y. Tsien, *Science*, 2002, **296**, 913-916.
2. M. B. Lutz, N. Kukutsch, A. L. J. Ogilvie, S. Rößner, F. Koch, N. Romani and G. Schuler, *J. Immunol. Meth*., 1999, **223**, 77-92.
3. S. Hoogendoorn, G. H. M. van Puijvelde, J. Kuiper, G. A. van der Marel, and H. S. Overkleeft, *Angew. Chem. Int. Ed.*, 2014, **53(41)**, 10975-8.
4. P. J. Peters, in Current Protocols in Cell Biology, John Wiley & Sons, Inc., 2001.
5. E. Bos, C. SantAnna, H. Gnaegi, R. F. Pinto, R. B. Ravelli, A. J. Koster, W. de Souza and P. J. Peters, *J. Struct. Biol*., 2011, **175**, 62-72.
6. J. R. Kremer, D. N. Mastronarde and J. R. McIntosh, *J. Struct. Biol*., 1996, **116**, 71-76.
7. J. Kuipers, T. J. van Ham, R. D. Kalicharan, A. Veenstra-Algra, K. A. Sjollema, U. Schnell, B. N Giepmans, *Cell Tissue Res*, 2015, **360(1)**, 61-70.
